# Abandoning mesh “overlap” in favor of “width” and its importance in open retromuscular midline incisional hernia repair: a nationwide database study

**DOI:** 10.1007/s10029-025-03423-7

**Published:** 2025-07-29

**Authors:** Mads Marckmann, Nadia A. Henriksen, Kristian S. Kiim

**Affiliations:** 1 Digestive Disease Center, Bispebjerg Hospital, University of Copenhagen, Bispebjerg Bakke 26, Copenhagen, 2400 Denmark; 2 Department of Surgery and Transplantation, Rigshospitalet, Copenhagen University Hospital, Copenhagen, Denmark

**Keywords:** Hernia repair, Hernia recurrence, Reoperation, Incisional hernia, Mesh repair, Mesh size

## Abstract

**Purpose:**

Recurrence after open incisional hernia repair remains an issue. Where the mesh preferably is placed in a retrorectus position, it is undetermined what the optimal mesh overlap is. This study aimed to assess the effect of mesh width on long-term recurrence after open incisional hernia repair.

**Method:**

The Danish Ventral Hernia Database was merged with The Danish National Patients Registry allowing a 100% follow-up. From 2011 to 2023 we included patients who underwent elective incisional hernia repair with vertical incision, retromuscular mesh placement, and linea alba reconstruction. Mesh and hernia size and repair type were registered. Kaplan-Meier plots showed cumulative incidences of operation for hernia recurrence over a 5-year period. Possible confounders were included in Cox proportional hazard and logistic regression analyses.

**Results:**

We included 1,539 patients. Mean (sd) age was 61.2 (12.4) years, 46.2% were females. Mean horizontal defect size was 8.4 (4.2) cm. Seventy-two (4.7%) patients underwent reoperation within 90 days and 112 (7.3%) developed recurrence (median follow-up 3.8 (IQR 1.8–6.1) years). Mesh width of 10–15 cm was associated with significantly decreased risk of operation for recurrence compared to both smaller and larger sizes (HR 0.38, CI 0.16–0.90, *P* = 0.029). Interestingly, fascial defect width was not associated with recurrence risk when adjusting for mesh width.

**Conclusion:**

A 10–15 cm mesh width is associated with lower risk of recurrence for patients undergoing elective open midline retromuscular incisional hernia repair: this “golden mean” should be of aim rather than “too little” or “as much as possible”.

## Introduction

Recurrence after open incisional hernia repair remains an issue with long-term rates after surgery ranging from 11 to 29% [1–5]. Well-acknowledged risk factors include surgical site occurrences and expertise in abdominal wall surgery, while the role of other factors such as demographic and intra-operative parameters are less well-determined [2, 3, 6]. These include mesh-related attributes and the comprehensive different options and combinations that mesh use implies: anatomic placement, type of filament, porosity, tensile strength, fixation method, mesh weight and size, and the clinical effects hereof. It is known that mesh repair lowers the risk of operation for recurrence compared to sutured repair, and there is a general consensus that in open hernia repair, it is preferred to place the mesh in the retrorectus position rather than onlay or intraperitoneally [5, 7–9].

The optimal mesh overlap has traditionally been considered to be 5 cm particularly when posterior component separation was introduced with its enhanced dissection possibilities enabling larger mesh overlap [10–12]. A systematic review from 2016 concluded that proper mesh overlap is a key determinant for recurrence after laparoscopic ventral hernia repair, but found no correlation to recurrence risk in open hernia repair [13]. Other studies reported a higher recurrence risk when using wider meshes without clear explanation [14, 15].

In recent years, focus has increasingly shifted towards the mesh/defect (M/D) ratio rather than mesh overlap itself with supportive studies leading to a change of recommendations in the 2019 update of *Guidelines for laparoscopic treatment of ventral and incisional abdominal wall hernias*: the 5-cm mesh overlap rule was abandoned and replaced by a statement that the mesh overlap should increase as the defect size increases [16–19]. Notably, these guideline changes were based on laparoscopic hernia repair studies without defect closure, and the recommendations were categorized as weak to moderate evidenced, thus they should not be uncritically applied to open hernia repairs. If the midline is closed in open hernia repair, the abdominal wall function is somehow restored, which earlier was shown to improve truncal function and quality of life [20]. Further, one could argue that this surgical step equalizes the starting point of the hernia repair by theoretically eliminating the effect of the hernia defect size. Hence, the terminology including “overlap” might be less relevant than simply just “size”.

This study hypothesized that an optimal mesh size with both a lower and upper limit exists based on the understanding that more dissection leads to more complications. We aimed to establish the importance and optimal horizontal size of the mesh in relation to subsequent recurrence development following open incisional hernia repair with midline closure.

## Methods

This was a study based on data from the Danish Ventral Hernia Database, where ventral hernia repairs have been registered since 2007 [21]. Several data are available from here including mesh and defect size, type of repair as well as layer for mesh placement and type of mesh. The Danish National Patients Registry holds information on all patients’ encounters with the public Danish health care system including reoperation codes [22]. Information from these two registries was merged enabling real-life data collection with a 100% follow-up.

### Inclusion criteria

In this study, all patients undergoing open, elective surgery for incisional hernia with vertical midline incision, retromuscular mesh placement, and linea alba reconstruction were included. Study period ran from January 1 2011 until February 28 2023.

### Variables

Age and sex were identified by the patients’ unique identification number. Charlson comorbidity index was categorized as none (0), mild (1), moderate (2), or severe (> 2). Horizontal and vertical hernia defect measurements were reported as numerical variables (mean with standard deviation), and for multivariable analysis categorized as < 4 cm, 4–8 cm, and > 8. Mesh width was grouped in four as < 10 cm, 10 - <15, 15–20, and > 20 cm. In the early years of the database it was not possible to specify type of component separation, thus for the current study this variable was categorized yes/no, and additionally subdivided in anterior component separation (ACS), transversus abdominis release (TAR), ACS + TAR, and unknown type. Readmission was defined as any hospital admission within 90 days after surgery. Reasons for readmission were registered via the Danish National Patients Registry including associated codes of diagnosis. Length of stay in hospital after surgery was registered numerically with median and range values. Short-term reoperation was defined as reoperation due to surgical complication within 90 days postoperatively. Recurrence was defined as operation for hernia recurrence during the follow-up period covering the time from index surgery to the time of data extraction.

For regression analyses of risk of hernia recurrence and risk of short-term reoperation we adjusted for the variables: age, sex, comorbidity, mesh width, primary or recurrent incisional hernia, and component separation (yes, no).

### Statistics

Continuous data were presented as mean (standard deviation) or median (interquartile range). Categorical data were presented as numbers (percentages). Data were compared across groups using the Chi-square test or the Mann-Whitney tests and p-values < 0.05 were considered statistically significant. The cumulative incidence of operation for hernia recurrence over a 5-year period was estimated using the Kaplan-Meier method. Variables likely to be associated with operation of recurrence and complications were included with Cox proportional hazard regression and logistic regression analysis, respectively. The statistics were performed using R software version 4.0.2 (R Foundation for Statistical Computing, Vienna, Austria).

### Ethics

This study was approved by the Danish Data Protection Agency REG-138-2018 and the Danish Hernia Database. The study was reported according to the STROBE statement for cohort studies [23].

## Results

**Fig. 1:**
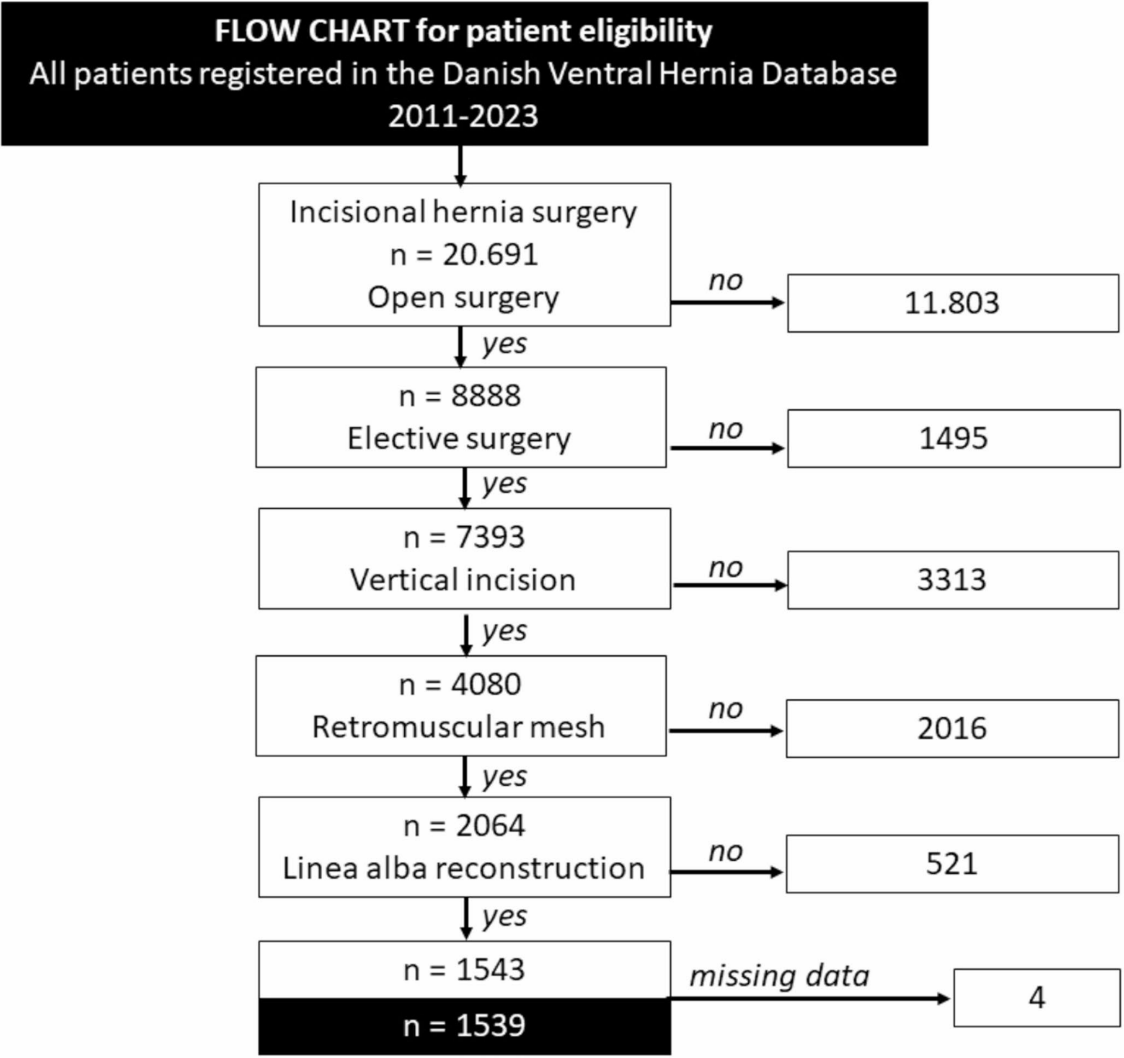
Flowchart

During the 12-year study period 20,691 patients underwent incisional hernia repair of which 8,888 were open. Of these, 1,539 patients were eligible for study inclusion (see flowchart, Fig. 1). Mean (sd) age was 61.2 (12.4) years and based on the national security number at the time of inclusion 46.2% were females. The mean (sd) horizontal defect size for the entire cohort was 8.4 (4.2) cm and median (IQR) length of stay was 4 days (0–8). A total of 333 (21.6%) patients were readmitted to the hospital within 90 days postoperatively.

Seventy-two (4.7%) patients required reoperation within 90 days after index surgery (Table 1). The characteristics of this group were comparable with the patients not reoperated regarding the variables sex, age, Charlson Comorbidity Index, horizontal defect size, and mesh width (*see* Table 2). Significantly more of the patients who required reoperation within 90 days postoperatively were readmitted compared to those who did not (75% vs. 19%) while the remaining 25% of the reoperations were performed during the initial hospital admission. Finally, 13 of the short-term reoperated patients subsequently underwent operation for hernia recurrence during study period, which were significantly more compared to the recurrence rate among patients who did not undergo short-term reoperation (18.1% vs. 6.7%, *P* = 0.001). See Table 2 for univariate analysis results regarding reoperation within 90 days.

**Table 1:** Demographics and overview of baseline data for the entire cohort of patients undergoing open elective incisional hernia repair

Variable		Total
Age (years)	*mean (sd)*	61.2 (12.4)
Sex	*female*	711 (46.2)
	*male*	828 (53.8)
Charlson Comorbidity Index	*0*	512 (33.3)
	*1*	236 (15.3)
	*2*	369 (24.0)
	*> 2*	422 (27.4)
Vertical defect size	*mean (sd)*	12.6 (6)
Horisontal defect size	*mean (sd)*	8.4 (4.2)
Horizontal defect size (cm)	*< 4*	225 (14.6)
	*4–8*	661 (42.9)
	*> 8*	653 (42.4)
Component separation (CS)	*No*	955 (62.1)
	*ACS*	71 (4.6)
	*TAR*	166 (10.8)
	*ACS + TAR*	37 (2.4)
	*Unknown type*	310 (20.1)
Mesh width (cm)	*0-9.99*	72 (4.7)
	10 - <15	360 (23.4)
	*15–20*	812 (52.8)
	*> 20*	295 (19.2)
Length of stay (days)	*median [IQR]*	4 [0–8]
	*missing*	28
Readmission within 90 days	*yes*	333 (21.6)
Reoperation within 90 days	*yes*	72 (4.7)
Follow-up (years)	*median (IQR)*	3.8 (1.8–6.1)
Recurrence during follow-up	*yes*	112 (7.3)

*IQR* interquartile range, *ACS* anterior component separation, *TAR* transversus abdominis release

**Table 2:** Results of univariate analysis stratified by reoperation of any cause within 90 days postoperatively (yes/no)

Variable		No (*n* = 1,467)	Yes (*n* = 72)	*P*
Age (years)	*mean (sd)*	61.2 (12.4)	60.9 (12.1)	0.866
Sex	*female*	677 (46.1)	34 (47.2)	0.954
	*male*	790 (53.9)	38 (52.8)	
Charlson Comorbidity Index	*0*	490 (33.4)	22 (30.6)	0.202
	*1*	229 (15.6)	7 (9.7)	
	*2*	353 (24.1)	16 (22.2)	
	*> 2*	395 (26.9)	27 (37.5)	
Horisontal defect size (cm)	*mean (sd)*	8.4 (4.2)	9.3 (4.2)	0.078
*categorized*	*< 4*	219 (14.9)	6 (8.3)	0.213
	*4–8*	631 (43.0)	30 (41.7)	
	*> 8*	617 (42.1)	36 (50.0)	
Mesh length (cm)	*mean (sd)*	26.1 (8.3)	28.6 (17.1)	0.022
Mesh width (cm)	*0-9.99*	71 (4.8)	1 (1.4)	0.202
	*10- <15*	346 (23.6)	14 (19.4)	
	*15–20*	766 (52.2)	46 (63.9)	
	*> 20*	284 (19.4)	11 (15.3)	
Component separation (CS)	*Yes*	548 (37.4)	36 (50.0)	0.042
Type of CS	*ACS*	68 (12.4)	3 (8.3)	0.772
	*TAR*	157 (28.6)	9 (25.0)	
	*ACS + TAR*	35 (6.4)	2 (5.6)	
	*Unknown type*	288 (52.6)	22 (61.1)	
Length of stay (days)	*median [IQR]*	4 [0–8]	4 [2–5]	0.555
	*missing*	28	0	
Readmission within 90 days	*yes*	279 (19.0)	54 (75.0)	< 0.001
Recurrence during follow-up	*yes*	99 (6.7)	13 (18.1)	0.001

*ACS* anterior component separation, *TAR* transversus abdominis release

A total of 112 (7.3%) patients underwent operation for recurrence during the study period and the variables age, sex and comorbidity distributed equally compared to patients that did not undergo operation for recurrence. In univariate analysis, horizontal defect > 8 cm was significantly associated with an increased risk of operation for recurrence with more than half of the patients in this category (52.7% vs. 41.6%, *P* = 0.019). Mesh width also differed significantly between the two groups: 14.3% of the patients who underwent operation for recurrence had a mesh width of 10-<15 cm compared to 24.1% in the non-recurrence group. Conversely, the patients with mesh widths of 0-<10 cm and > 20 cm had a higher proportion in the recurrence group compared to the non-recurrence group (7.1 vs. 4.5% and 28.8 vs. 18.4%, respectively) (*P* = 0.009). The 90-day readmission rate was higher in recurrence group (32.1% vs. 20.8% *P* = 0.007) as was the 90-day reoperation rate (13% among patients with recurrence vs. 4.1% among the patients without, *P* = 0.001) (Table 3).

**Table 3:** Results of univariate analysis stratified by reoperation for hernia recurrence during long-term follow-up

Variable		Patients not operated for recurrence (*n* = 1,427)	Patients operated for recurrence (*n* = 112)	*P*
Age (Years)	*mean (sd)*	61.3 (12.4)	59.1 (12.4)	0.063
Sex	*female*	661 (46.3)	50 (44.6)	0.807
	*male*	766 (53.7)	62 (55.4)	
Charlson Comorbidity Index	*0*	474 (33.2)	38 (33.9)	0.975
	*1*	219 (15.3)	17 (15.2)	
	*2*	344 (24.1)	25 (22.3)	
	*> 2*	390 (27.3)	32 (28.6)	
Horizontal defect size (cm)	*mean (sd)*	8.4 (4.2)	9 (4.9)	0.140
* categorized*	*< 4*	206 (14.4)	19 (17.0)	0.019
	*4–8*	627 (43.9)	34 (30.4)	
	*> 8*	594 (41.6)	59 (52.7)	
Mesh length (cm)	*mean (sd)*	26.2 (8.3)	27.2 (15.2)	0.244
Mesh width (cm)	*0-9.99*	64 (4.5)	8 (7.1)	0.009
	*10- <15*	344 (24.1)	16 (14.3)	
	*15–20*	756 (53.0)	56 (50.0)	
	*> 20*	263 (18.4)	32 (28.6)	
Component separation (CS)	*Yes*	536 (37.6)	48 (42.9)	0.312
Type of CS	*ACS*	69 (12.9)	2 (4.2)	0.002
	*TAR*	158 (29.5)	8 (16.7)	
	*ACS + TAR*	37 (6.9)	0 (0.0)	
	*Unknown type*	272 (50.7)	38 (79.1)	
Length of stay (days)	*median [IQR]*	4 [0–4]	5 [1–6]	0.047
	*missing*	25	3	
Readmission within 90 days	*Yes*	297 (20.8)	36 (32.1)	0.007
Reoperation within 90 days	*Yes*	59 (4.1)	13 (11.6)	0.001

*ACS* anterior component separation, *TAR* transversus abdominis release

**Fig. 2:**
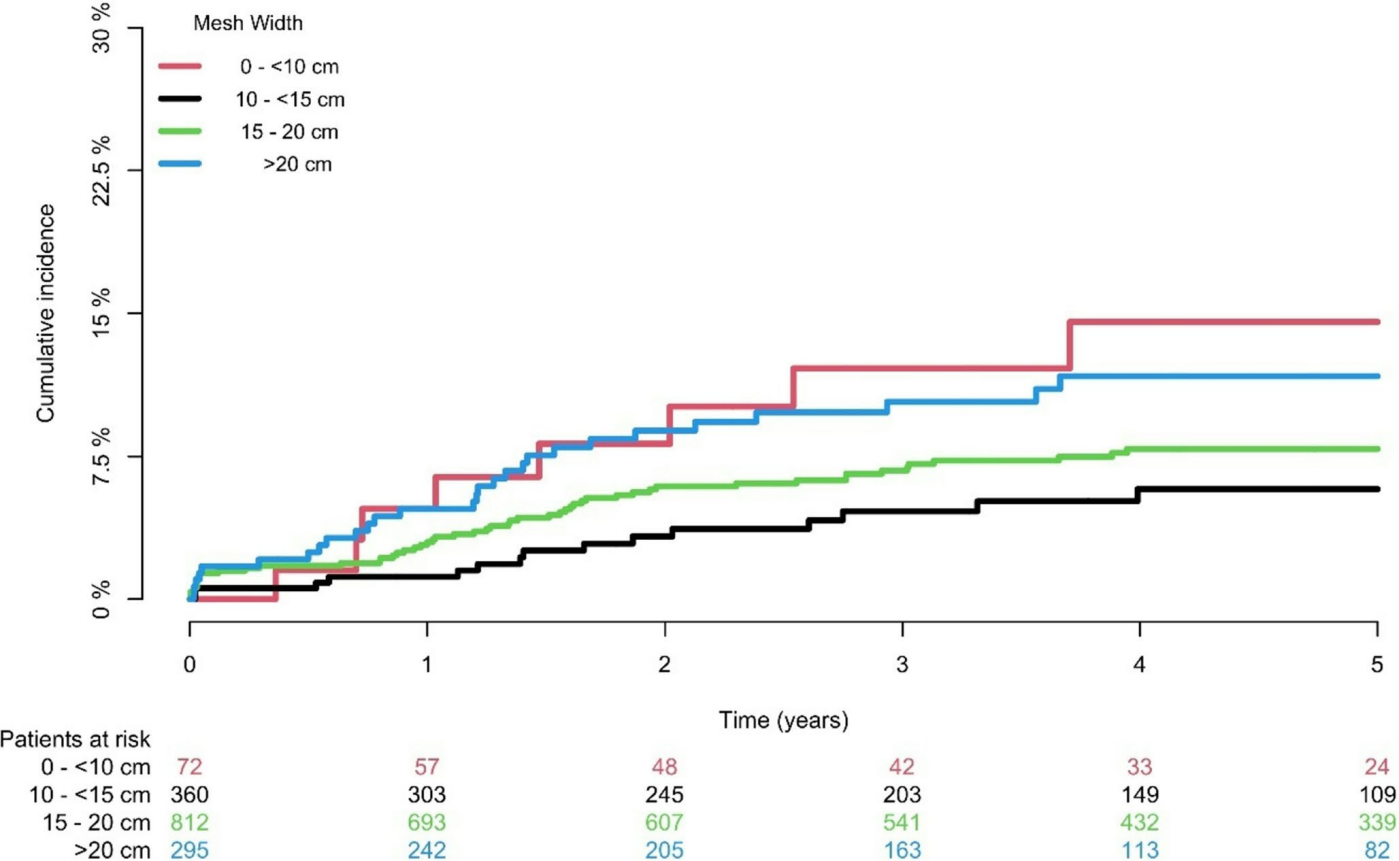
Cumulative incidence curves for reoperation for hernia recurrence by mesh width after index incisional hernia repair. *P* = 0.006

The cumulative incidences of operation for recurrence for the entire cohort after one year, three years, and five years were: 2.98% (95% CI 2.10–3.86), 7.18% (5.77–8.59), and 8.41% (95% CI 6.84–9.98), respectively. Median (IQR) follow-up time were 3.8 (1.8–6.1) years. Kaplan-Meier curves showed that for a mesh width of 10-<15 cm, the absolute risk of operation for recurrence was 5.78% (95% CI 2.84–8.71), which was significantly lower than for the other groups of 0–10 cm, > 15–20 cm, and > 20 cm: 14.54% (95% CI 5.04–24.04), 7.89% (95% CI 5.84–9.94), and 11.69% (95% CI 7.51–15.87), respectively (log-rank *P* = 0.006) (Fig. 2). Multivariable Cox regression showed a lower hazard for operation for recurrence for a mesh width of 10-<15 cm (HR 0.38 95% CI 0.16–0.90 *P* = 0.029) adjusted for confounders such as horizontal defect size, age, comorbidity, and component separation. Regarding risk of 90-day reoperation, no significant correlations or trends associated with mesh width were detected (Tables 4 and 5).

**Table 4:** Cox proportional hazards multivariable regression analysis of the risk for reoperation for hernia recurrence

Variable		HR	95% CI	*P*
Sex	Female	Ref		
	Male	1.00	0.68–1.46	0.997
Age		0.99	0.97-1.00	0.129
CCI	0	Ref		
	1	1.09	0.61–1.96	0.775
	2	1.16	0.68–1.98	0.581
	> 2	1.27	0.76–2.12	0.357
Horizontal defect size		1.02	0.97–1.07	0.422
Mesh width	0-9.99	Ref		
	10 - <15	0.38	0.16–0.90	0.029
	15–20	0.55	0.25–1.21	0.137
	> 20	0.90	0.37–2.16	0.806
Index surgery	Non-recurrence	Ref		
	Recurrence	1.76	1.15–2.67	0.009
Component separation	Yes	Ref		
	No	1.09	0.70–1.68	0.707

*CCI *Charlson comorbidity Index, *HR* Hazard Ratio

**Table 5:** Logistic regression analysis of the risk for reoperation within 90 days after index incisional hernia repair

Variable		OR	95% CI	*P*
Sex	Female	Ref		
	Male	0.92	0.57–1.49	0.726
Age		0.99	0.97–1.01	0.456
CCI	0	Ref		
	1	0.64	0.27–1.55	0.325
	2	1.04	0.52–2.07	0.917
	> 2	1.50	0.81–2.79	0.202
Horizontal defect size		1.03	0.96–1.09	0.444
Mesh width	0-9.99	Ref		
	10 - <15	2.25	0.29–17.70	0.442
	15–20	3.03	0.40-23.04	0.283
	> 20	1.55	0.18–13.01	0.689
Index surgery	Non-recurrence	Ref		
	Recurrence	1.06	0.55–2.01	0.869
Component separation	Yes	Ref		
	No	0.62	0.36–1.08	0.095

*CCI *Charlson comorbidity Index, *or* odds Ratio

## Discussion

**Fig. 3:**
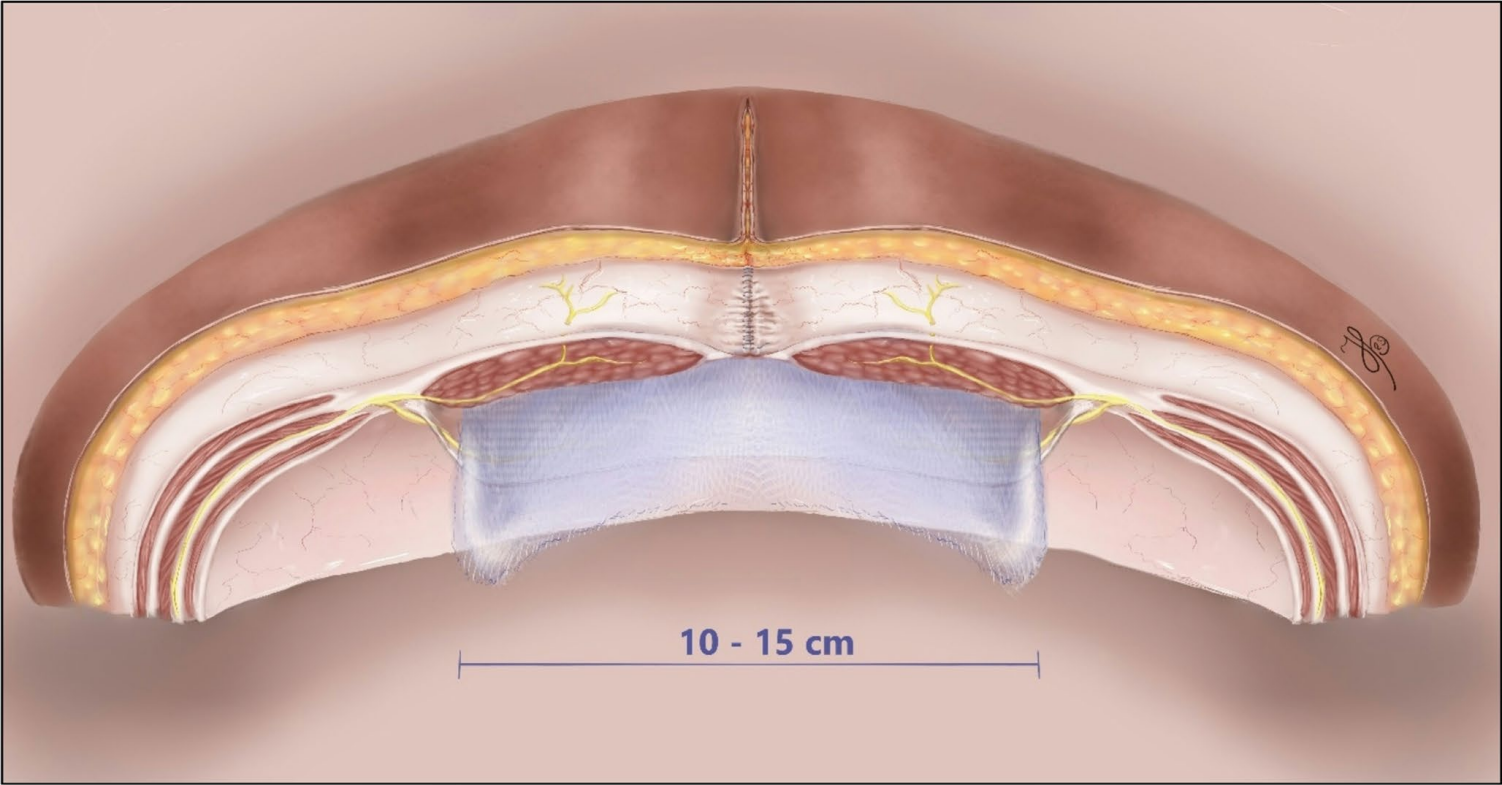
Illustration of a transverse view of the abdominal wall layers showing the optimal mesh width in the retrorectus position with a closed fascial defect. This size was significantly associated with a lower risk of long-term recurrence. *Illustration by © Helene Perregaard*,* MD*

This nationwide database study found that for patients undergoing elective open midline retromuscular incisional hernia repair, a mesh width of 10–15 cm was associated with a significantly reduced risk of long-term hernia recurrence compared to both narrower and wider meshes (Fig. 3). The association between mesh width and recurrence risk was significant regardless the size of the horizontal defect, a result that challenges the idea of “increasing mesh size with increasing defect size”, which earlier studies in laparoscopic settings highlighted as important [16–19]. This also suggests that even though extensive dissection is possible, it is not necessarily needed nor advantageous. Notably, all selected patients in the current study underwent midline closure, which has proved to lower the risk of seroma formation, surgical site adverse events, as well as optimize truncal function and quality of life postoperatively [20, 24]. When the midline is sutured in open mesh repair, the hernia defect is closed, i.e., the abdominal wall is restored, the gap is filled. In the light of this and the results of the study, the term “overlap” does not seem intuitive or appropriate to use rather than simply just mesh “width”, and perhaps should be saved for laparoscopic hernia repairs where the defect is not closed and where sufficient overlap is critical to reduce the risk of recurrence, as LeBlanc found in a systematic review [13].

Pragmatically, to compare our results with earlier findings and temporarily accept the “overlap” term, a 10–15 cm width could be converted to a 5–7.5 cm overlap after defect closure. This relatively specific size both supports the old dogma of the “5-centimeter rule”, but also suggests that 4 cm is not enough as it implies a higher risk of hernia recurrence despite what was earlier reported when Rosen and Pauli described the procedure of open ventral hernia repair with component separation [25]. This interval also sets an “upper overlap limit” as mesh widths of 15 cm and above showed an increase in recurrence risk, a notion supported by few earlier studies [14, 15]. A key message from the findings of this study is that dissection can be overdone and should not be a goal itself. Importantly, however, anatomical conditions such as the size of the retrorectus pocket surely varies between individuals, thus, very narrow rectus muscles might be insufficient to accommodate a mesh width of 10 cm, in which case further dissection—i.e. posterior component separation—could be indicated [26]. Regarding direct comparison with studies on open retrorectus mesh repair, it currently seems impossible due to a lack of literature. In 2020 the European Hernia Society and the American Hernia Society published guidelines on the preferred mesh overlap for open umbilical or epigastric repair suggesting 3 cm for defects of 1–4 cm, however, this recommendation solely regarded preperitoneal mesh repair and was tainted with the lowest level of both evidence and recommendation strength [27]. In this study we included horizontal mesh size and omitted vertical mesh size in multivariate analysis: this distinction was made with referral to the physiologic conditions of the abdominal wall, in which transverse forces drive the tensile strength, why this direction has been suggested as where the orientation for the strongest axis of a mesh should be, whereas the most compliant axis should be in the longitudinal direction [28].

The overall 5-year recurrence rate of 8.4% in this cohort was lower than what an earlier large database study reported (12.7%), however, this could be explained by the fact that they included all mesh positions of which the minority were retrorectus [5]. In this context, it should be noted that the recurrence rates reported in this study only included the cases that were reoperated, while the clinical recurrence rate inherently is higher. The findings of this study showed that patients initially short-term reoperated due to complications had a significantly higher risk of subsequent hernia recurrence, supporting earlier evidence [29].

The Danish Ventral Hernia Database is a mandatory registry, but limitations include that it cannot be guaranteed that all cases eligible for inclusion were in fact registered, however the registration rates are high [21]. Also, since data were not randomized, a risk of selection bias is present. Reportedly, smoking is a risk factor for long-term hernia recurrence and together with obesity increase the risk of short-term readmission and re-operation, unfortunately, in this study we were unable to evaluate these factors as the numbers of missing data were too high to yield relevant conclusions [30–32]. Finally, earlier findings indicated that using lightweight meshes increases the risk of hernia recurrence, however, we were not able to make this analysis due to unavailability of data [2].

## Conclusions

The results of this study show that for patients undergoing elective open midline retromuscular incisional hernia repair with midline closure, the horizontal size of the applied mesh does play an important role regardless the size of the hernia defect. If attainable, a “golden mean width” of 10–15 cm should be of aim as this interval lowers the risk of subsequent operation for hernia recurrence, and therefore is more appropriate than both “too little” and “as much as possible” along with the extent of dissection. Also, the results of this study add a terminological revision, suggesting that for open hernia mesh repairs where the defect is closed, the term “mesh overlap” should be abandoned and replaced with “mesh width” to reflect the structural physiological implications of the surgical procedure more accurately. Randomized trials are warranted to provide supporting evidence.

## Data Availability

Data will be available on request.
